# Epidemic Potential for Local Transmission of Zika Virus in 2015 and 2016 in Queensland, Australia

**DOI:** 10.1371/currents.outbreaks.73d82b08998c6d729c41ef6cdcc80176

**Published:** 2016-12-13

**Authors:** Elvina Viennet, Gina Mincham, Francesca D. Frentiu, Cassie C. Jansen, Brian L. Montgomery, David Harley, Robert L.P. Flower, Craig R. Williams, Helen M. Faddy

**Affiliations:** Research and Development, Australian Red Cross Blood Service, Kelvin Grove, Queensland, Australia; Centre for Population Health Research, University of South Australia, Adelaide, South Australia, Australia; Institute of Health and Biomedical Innovation & School of Biomedical Sciences, Queensland University of Technology, Kelvin Grove, Queensland, Australia; Metro North Public Health Unit, Metro North Hospital and Health Service, Windsor, Queensland, Australia; Metro South Public Health Unit, Metro South Hospital and Health Service, Brisbane, Queensland, Australia; Research School of Population Health, The Australian National University, Australian Capital Territory, Australia; Research and Development, Australian Red Cross Blood Service, Kelvin Grove, Queensland, Australia; Centre for Population Health Research, University of South Australia, Adelaide, South Australia, Australia; Research and Development, Australian Red Cross Blood Service, Kelvin Grove, Queensland, Australia

## Abstract

Introduction: Zika virus could be transmitted in the state of Queensland, Australia, in parts of the state where the mosquito vectors are established.

Methods: We assessed the epidemic potential of Zika in Queensland from January 2015 to August 2016, and estimate the epidemic potential from September to December 2016, by calculating the temperature-dependent relative vectorial capacity (rVc), based on empirical and estimated parameters.

Results: Through 2015, we estimated a rVc of 0.119, 0.152, 0.170, and 0.175, respectively in the major cities of Brisbane, Rockhampton, Cairns, and Townsville. From January to August 2016, the epidemic potential trend was similar to 2015, however the highest epidemic potential was in Cairns. During September to November 2016, the epidemic potential is consistently the highest in Cairns, followed by Townsville, Rockhampton and Brisbane. Then, from November to December 2016, Townsville has the highest estimated epidemic potential.

Discussion: We demonstrate using a vectorial capacity model that ZIKV could have been locally transmitted in Queensland, Australia during 2015 and 2016. ZIKV remains a threat to Australia for the upcoming summer, during the Brazilian Carnival season, when the abundance of vectors is relatively high. Understanding the epidemic potential of local ZIKV transmission will allow better management of threats to blood safety and assessment of public health risk.

## Introduction

In 2015, Zika virus (ZIKV) emerged throughout the Americas and in February 2016, the World Health Organization declared ZIKV a public health emergency of international concern.

ZIKV is principally transmitted from infected *Aedes aegypti* and *Aedes albopictus* mosquitoes, vectors of dengue and chikungunya viruses. ZIKV can also be transmitted from mother to fetus, through sexual contact and via blood transfusion^1^^,^ 2. Up to eighty percent of infected people are asymptomatic^3^. In adults, the illness is usually mild, and common symptoms include fever, rash, joint pain and conjunctivitis, lasting for up to one week. However, ZIKV can cause serious birth defects when acquired during pregnancy^4^.

In north and central Queensland, Australia, where *Ae. aegypti* is present, ZIKV could be locally transmitted if imported. Between 2012 and 7 October 2016, 36 of the 76 confirmed ZIKV infections in travelers notified nationally were reported from Queensland^5^. In response to the emerging public health risk, diagnostic services for ZIKV and preventative vector control have been strengthened in the north of the state. Local transmission has not yet been recorded; however due to the high asymptomatic rate, cryptic transmission is possible. We retrospectively visualize the epidemic potential of Zika in four cities in Queensland (Brisbane, Cairns, Rockhampton and Townsville) from January 2015 to August 2016, and estimate the epidemic potential from September to December 2016. This was temporally relevant due to the risk of ZIKV importation from the Rio 2016 Olympic and Paralympic Games^6^.

## Materials and Methods

**Study Area**

Four cities in Queensland were selected: Cairns, Townsville and, Rockhampton, which share current potential risk of Zika transmission as *Ae. aegypti* is well established; and, Brisbane, the state capital, where there is no evidence of *Ae. aegypti* since the 1950s^7^. However, *Ae. aegypti* is present in regions of Queensland that are only a few hundred km north of Brisbane (e.g. township of Gin-Gin).

**Data Set**

Monthly minimum and maximum temperatures (°C) from January 2015 to August 2016 inclusive, were retrieved from the Australian Bureau of Meteorology^8^. Monthly average mean temperature was calculated (Fig. 1).

Monthly predictions of the maximum and minimum temperature medians for September to December 2016 based on historical values were used to estimate the corresponding mean temperature medians^9^ (Fig. 1).

**Monthly average temperature in Queensland cities, Australia, January 2015 - December 2016.**
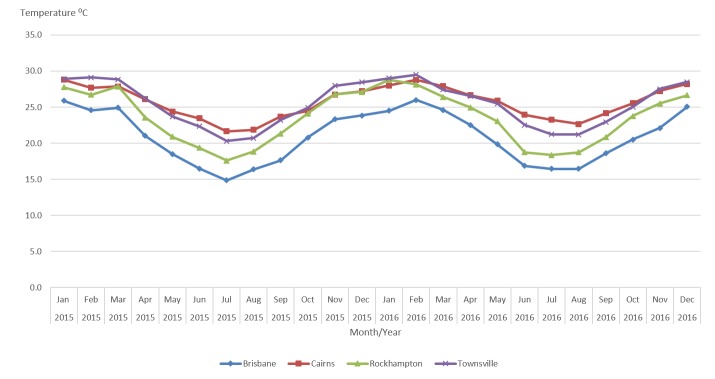

**Estimation of Zika Epidemic Potential**

The relative vectorial capacity (*rVc*) represents the vector’s ability to spread disease among humans relative to the vector-to-human population ratio^10^ and is defined as:

\begin{equation*}rVc = a^{2} (T)  \times b_{h} (T)\times b_{m} (T)\times \frac{e^{-\mu _{m}(T)\times  }n(T)}{\mu_{m}(T)}\end{equation*}

with *a*) the average daily vector biting rate; *T*) temperature;* b_h_*) the probability of vector-to-human transmission per bite; *b_m_*) the probability of human-to-vector infection per bite; *µm*) the vector mortality rate; *n*) the duration of extrinsic incubation period (Table 1).

**Table 1: Parameters used to estimate the relative vectorial capacity of Zika in Queensland, Australia:** 

Parameters	Description	Estimate/equations	Relationship	Source
a(T)	average daily vector biting rate (number of bites per day)	= 0.0043T + 0.0943 with 21◦C ≤ T ≤ 32◦C	Virus-independent; Temperature-dependent	10
b_m_(T)	probability of human-to-vector infection per bite	= 0.0729T – 0.9037 if 12.4°C ≤ T ≤ 26.1°C	Virus-and temperature-dependent	10
		= 1 if 26.1°C < T < 32.5°C		
		= 0.57		13
b_h_(T)	probability of vector to human transmission per bite	= 0.001044T(T – 12.286)x(32.461-T)^1/2 ^ for 12.286°C ≤ T < 32.461°C	Virus-and temperature-dependent	10
		= 0.27		13
n	duration of extrinsic incubation period (day)	= 7	Virus-and temperature-dependent	11 ^,^ 12
		= 10		14
		= 4 + e ^5.15-0.123T^		10
µ_m_(T)	Daily vector mortality rate (number of deaths in mosquito population per day)	= 0.8692 – 0.1590T + 0.01116T^2^ – 3.408 x 10^-4^T^3 ^+ 3.80 X 10^-6^T^4^	Virus-independent; Temperature-dependent	10

The last has been estimated to be seven (ranging from five to ten)^11^^,^ 12 ^,^ 13 and to ten days^14^. *b_h_*, and *b_m_* have recently been estimated for ZIKV and Australian *Ae. aegypti* mosquitoes^13^. Dengue virus (DENV) is also transmitted by *Ae. aegypti* mosquitoes, for which the other parameters and their relationship to temperature have been well described^10^^,^ 15 ^,^ 16. Therefore, we adapted a temperature-driven model for DENV transmission by *Ae. aegypti* 10 ^,^ 15 ^,^ 16, with estimated values of *n*, *b_h_*, and *b_m_* for ZIKV to assess and compare the Zika epidemic potential over space and time^10^.

We performed three relative vectorial capacity calculations, equation 1 and 2 with a varying *n*, and equation 3, as a comparison, with all parameters specific to DENV.

***Equation 1***: Formula 1) with the average *n* equal to 7, based on Chouin-Carneiro et al. (2016), Li et al. (2012), and Hall-Mendelin et al. (2016). Hereafter referred to as* rVc_7_*.****

***Equation 2***: Formula 1) with the average *n* equal to 10, based on Boorman and Porterfield (1956). Hereafter referred to as *rVc_10_*.

***Equation 3***: Formula 1) based on the DENV-specific parameters from Liu-Helmersson et al. (2014). Hereafter named *rVc_(T)dengue_*.

Monthly epidemic potentials and range from equations 1, 2, and 3 were calculated for each city. For January 2015-August 2016, mean maximum and minimum temperatures each month were used for calculations of* rVc*. Forecasts for future monthly epidemic potential in September-December 2016 were estimated as described above. We then compared the results from equations 1 and 2 using an analysis of variance (ANOVA), with the null hypothesis being that the mean of the relative vectorial capacity remains the same for both scenarios.

**Threshold Value for the Epidemic Potential**

The threshold value *rVc^* for an epidemic to take place is defined by:

\begin{equation*}rVc^{\Lambda} = \frac{1}{T_{h} \times m}\end{equation*}

Although analyses of the infectious period (*Th*) for ZIKV are still ongoing, a recent study of 297 PCR-confirmed clinical cases in French Polynesia estimated *Th* to last for four to seven days^17^^,^ 18. Using an average value of *Th* of 5.5 days, and assuming that the human-to-mosquito ratio, *m*, equals 1, the *rVc*^ must be larger than 0.18 [0.14; 0.25] per day for a Zika outbreak to occur. As an uncertainty analysis, we used the variability range of the infectious period [4, 7] to estimate the range of the threshold value for the epidemic potential.

## Results

The resulting *rVc* from equations 1 (*rVc7*), 2 (*rVc10*) and 3 (*rVc(T)dengue*) are presented in Fig. 2. The *rVc(T)dengue* is consistently higher than *rVc7* and *rVc10* throughout the study period in Townsville and Cairns (Fig. 2).

**Comparison of epidemic potentials calculated using three relative vectorial capacity estimates of mosquitoes to transmit Zika virus in Queensland cities, Australia, January 2015 - December 2016.**
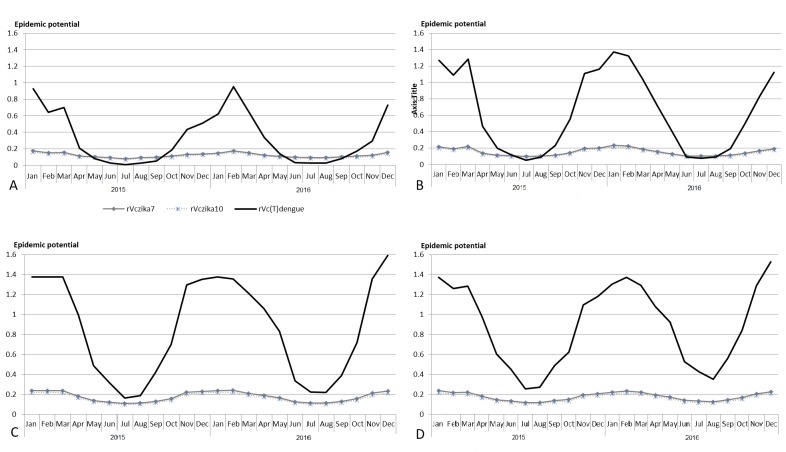
A) Brisbane, B) Rockhampton, C) Townsville, D) Cairns; rVc: relative vectorial capacity; rVc7: when the extrinsic incubation equal 7; rVc10: when the extrinsic incubation equal 10; rVc(T) corresponds to Liu-Helmersson et al. (2014) calculation with temperature as a variable for each parameters

At α = 0.05, p_value = 0.139 and* F critical* is larger than *F*, therefore we did not reject the null hypothesis. The difference between the mean *rVc7* and *rVc10* was, therefore, not significantly different from zero. The equations are thus interchangeable, so we used *rVc7* in the following analyses.

Through 2015, we estimated an overall relative vectorial capacity (*rVc7*) of 0.119 in Brisbane, 0.152 in Rockhampton, 0.170 in Cairns, and 0.175 in Townsville. Throughout 2015, the lowest *rVc7* was estimated for Brisbane. When compared with Townsville, the estimated *rVc7* in Cairns was lower from January to April and October to December 2015, but higher during the rest of the year (Fig. 3).

**Estimation of the relative vectorial capacity of mosquitoes to transmit Zika virus in Queensland cities, Australia, from January 2015 - August 2016 and forecast from September to December 2016.**
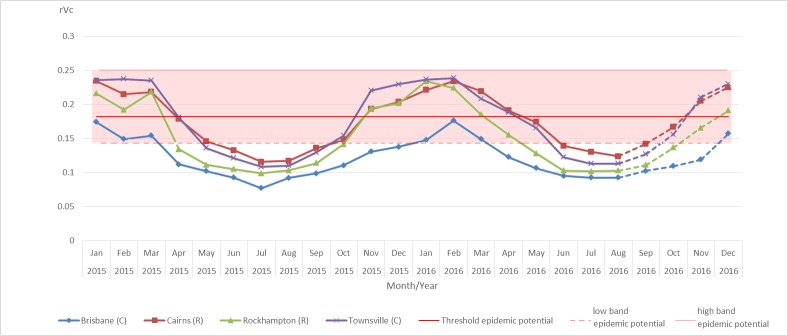
Lines: Estimation from observed temperatures; dash: Estimation from predicted temperatures The relative vectorial capacity was estimated by using rVc7. Red line shows mean of the threshold value of the epidemic potential; shaded region shows its variability range

From January to August 2016, the epidemic potential trend was similar to 2015 with the highest epidemic potential in Cairns (Fig. 3), and an estimated *rVc7* of 0.123 in Brisbane, 0.154 in Rockhampton, 1.173 in Townsville, and 1.179 in Cairns. From September to November 2016, the monthly estimated epidemic potential based on the mean temperature median predictions was consistently the highest in Cairns, followed by Townsville, Rockhampton and Brisbane (Fig. 3), with estimates of 0.171, 0.164, 0.138, and 0.110, respectively. Then, from November to December 2016, Townsville has the highest estimated epidemic potential.

With a threshold value for a Zika outbreak fixed at *rVc* = 0.18 day^-1^, and assuming suitable transmission conditions without vector control programs, Cairns, Townsville and, to a lesser extent, Rockhampton, would have been suitable for ZIKV transmission during the warmer seasons in 2015 and 2016. When the threshold value is equal to 0.14, (*Th* equal to seven days), Brisbane would also become suitable for ZIKV transmission during the warmer months and Rockhampton, Townsville and Cairns, would have a longer potential transmission season for ZIKV. If, however, *Th* is equal to four days (*rVc* = 0.25 day^-1^), none of these cities would be suitable for ZIKV transmission. When the threshold value *rVc* varies within [0.14; 0.25], the suitability for each city to transmit ZIKV might change (see Fig. 3).

## Discussion and Conclusions

Zika is a significant global public health issue. We demonstrate that, based on an average infectious period of 5.5 days^17^^,^ 18, Zika virus transmission by vector mosquitoes could have occurred in Cairns, Townsville and Rockhampton during the warmer months in 2015 and 2016, with Brisbane unsuitable for transmission. However, this changes when we take a lower epidemic potential threshold, based on a longer infectious period. Except for Brisbane where the vector is not yet established, these results are consistent with the epidemic potential of dengue virus in Cairns and Townsville.

While estimates are preliminary, these analyses point to the importance of further investigations of the infectious period as well as the infection and transmission rates of ZIKV and Australian mosquitoes (*Ae. aegypti* and* Ae. albopictus*) to allow more accurate estimates of the epidemic potential for ZIKV in Australia.

The Rio de Janeiro 2016 Olympics (5–21 August 2016) and the Paralympic Games (7–18 September 2016) were the two most prominent mass gathering events to take place in the Americas since the emergence of ZIKV in this region. A large number of visitors attended these events^6^. An upcoming event in Brazil that also attracts tourists from Australia is the Rio de Janeiro Carnival (24-28 February 2017), a period where mosquito abundance is highest (in Brazil as well as Queensland).

Our study considered only the risk posed by vector-mediated transmission and did not include consideration of the potential for sexual transmission^19^^,^ 20 ^,^ 21 which may occur across Australia, irrespective of the presence or absence of suitable vectors. Importantly, a recent study has shown that while sexual transmission increases the risk of infection and epidemic size (number of cases), by itself it may not initiate or sustain an outbreak^22^. Thus it is likely that vector-borne transmission is, by far, the greatest concern to vulnerable locations in Queensland.

More importantly, ZIKV poses a particular challenge in that 80% of cases have no symptoms, and thus would not be detected by a syndromic surveillance system^23^. We were particularly concerned with the prospect that infected travelers from the 2016 Rio Olympics or Paralympics games arriving in Cairns or Townsville might carry Zika, and our projections show that, in this event, epidemic transmission would have been low in winter, but possible during the warmer months in North Queensland.

Vector-borne transmission of ZIKV is expected to have a similar seasonal pattern to that of dengue and chikungunya, with lower transmission in winter. Nonetheless, the potential for vector-mediated transmission in the absence of overt cases due to asymptomatic infections will likely require a shift of focus for health authorities from emergency response currently effective for dengue case notifications toward broad-scale preventative programs, and emerging control methodologies (for example, *Wolbachia*, which is currently being released in Cairns and Townsville regions as a research field trial)^24^. The frequency of ZIKV importations into South East Queensland suggests that an investment in more comprehensive vector surveillance would be warranted to confirm the apparent absence of *Ae. aegypti* from Brisbane since the 1950s, and detect any incursions from the nearby endemic regions of Queensland. ZIKV transmission through blood transfusion is possible. Therefore, this virus poses a threat directly to blood safety, and indirectly on donor attendance.

This study has significant implications, highlighting the importance of adapting timely preventive measures and case management to an emerging arbovirus and underscores the public health challenges associated with mass gathering events involving the international movement of attendees returning to vulnerable cities. Although at this stage, the epidemic potential for early 2017 could not be forecast, it is likely that it will follow a similar trend to 2015-2016. Attendees of the Carnival in Brazil (along with other travelers visiting other zika-affected areas), who are traveling to Queensland where the vector is established, should follow the precautions and recommendations issued by their national health authorities. In north and parts of central Queensland where the vectors are present, immediate reporting of clinically suspected cases of ZIKV infection to local public health units is critical.

## Corresponding Author

Elvina Viennet: eviennet@redcrossblood.org.au

## Competing Interests

The authors have declared that there are no competing interests.

## Data Availability

All relevant data have been made available in the figshare repository (DOI: 10.6084/m9.figshare.3993981).
